# Oxygen-Mediated Molecular Mechanisms Involved in Intestinal Ischemia and Reperfusion Injury

**DOI:** 10.3390/ijms26178398

**Published:** 2025-08-29

**Authors:** Paraschos Archontakis-Barakakis, Theodoros Mavridis, Athanasios Chalkias

**Affiliations:** 1 Department of Medicine, Redington-Fairview General Hospital, Skowhegan, ME 04976, USA; 2 Department of Medicine, Tufts University School of Medicine, Boston, MA 02111, USA; 3 Department of Neurology, Tallaght University Hospital (TUH), The Adelaide and Meath Hospital Incorporating the National Children’s Hospital (AMNCH), D24NR0A Dublin, Ireland; mavridismdr@gmail.com; 4 Institute for Translational Medicine and Therapeutics, Perelman School of Medicine, University of Pennsylvania, Philadelphia, PA 19104, USA; thanoschalkias@yahoo.gr; 5 Outcomes Research Consortium^®^, Houston, TX 77030, USA; 6 Department of Critical Care Medicine, General Hospital of Piraeus “Tzaneio”, 18536 Piraeus, Greece

**Keywords:** molecular mechanisms, intestinal hypoxia, ischemia–reperfusion injury

## Abstract

The gastrointestinal tract is affected by multiple ailments that manifest with similar chemical, subcellular, and cellular changes, such as those in intestinal ischemia–reperfusion injury (IRI). The main chemical changes that are described under IRI conditions include the depletion of oxygen available for normal metabolism and the abundant production and increase in intracellular and extracellular concentrations of hydrogen peroxide and other reactive oxygen species (ROS). The enzymes causing this accumulation are xanthine dehydrogenase turning into xanthine oxidase, nicotinamide adenine dinucleotide phosphate oxidase, and nitric oxide synthase. The cellular changes revolve around an oxygen-sensing system that is responsive to varying oxygen levels, which has Hypoxia-Inducible Factors (HIFs) at its base. HIFs are transcription factors, the intracellular concentrations of which significantly increase under hypoxic conditions. Upon activation, they alter the expression of gene sets to ensure appropriate cellular adjustment to the hypoxic and IRI environment. Despite the primary regulation of the system involving oxygen, it is interconnected with multiple other subcellular and cellular functions. Thus, it represents a linchpin control mechanism of cellular adaptation. The effect of HIF activation in intestinal cells aims at preserving the structural integrity of the intestinal lining. The effect in different subtypes of leucocytes aims at immune system activation to protect against previously luminally located and subsequently invading pathogens and toxins. All in all, the HIF system is an integral part of cellular and tissue compensation against intestinal IRI.

## 1. Introduction

The gastrointestinal (GI) tract is tasked with the absorption of all necessary nutrients to maintain homeostasis. At the same time, it is heavily contaminated intraluminally by bacteria that participate in various digestive processes. The size of the system and its flora make it vulnerable to injury caused by a long list of both topical and systemic ailments.

A substantial portion of those ailments have common pathophysiological characteristics, collectively known as ischemia/reperfusion injury (IRI). This deleterious process stems from oxygen deprivation and misutilization, exhibits common tissue-level manifestations, and develops in a similar fashion despite very different etiologies. IRI is complex and affects cellular chemistry, cellular function and metabolism, and organ structure and viability. The buildup of substantial damage can even lead to a disseminated response with different levels of intensity, such as bacteremia, sepsis, systemic inflammatory response syndrome, and multiple organ dysfunction syndrome [1,2,3,4,5,6,7].

Extensive translational research has shown that the diminished oxygen availability for core cellular mechanisms and the impaired utilization of available oxygen represent a linchpin of the associated local and systemic responses [8]. This review aims to describe the molecular mechanisms associated with oxygen that are involved in intestinal IRI.

## 2. Natural History of IRI

IRI starts with the establishment of impaired blood and oxygen supply to the entirety or just a portion of the GI tract. The listing of specific causes, the description of clinical syndromes, and the analysis of IRI manifestations within those syndromes fall outside the scope of this review. Briefly though, the main pathophysiologic mechanisms include large-sized arterial thrombosis, mainly represented by acute mesenteric ischemia, large-sized venous thrombosis, primarily manifested as mesenteric venous thrombosis, GI hypoperfusion, and immune infiltration of the intestine, e.g., in patients with intestinal transplant rejection [7,9,10,11,12,13,14]. Of note, IRI has been clearly identified within the context of seemingly unrelated medical conditions such as volvulus [11,15].

The ischemic phase of IRI starts immediately after the establishment of oxygen deprivation or misutilization. The cellular and tissue changes identified in the initial stages of the process are generally reversible. There is no specific time frame that dictates whether this threshold of reversibility is crossed; a duration of injury ranging from minutes to hours usually incorporates the reversible period and is closely related to the ability of capillaries to adjust to micro-ischemic changes [16,17,18]. Except for timing, there is also no exact oxygen concentration at which this shift takes place. Thus, ischemia represents a continuum of injury and cellular and tissue responses happening simultaneously. If hypoperfusion and oxygen deprivation continue, the galloping cascade of ischemia will lead to irreversible changes [19]. Reperfusion, the second phase of IRI, starts with the restoration of perfusion and oxygenation to the affected organ or tissue. Although this is the first step towards recovery, the concomitant activation of multilevel molecular and systemic mechanisms may aggravate intestinal injury. This reperfusion alongside ischemia generates an even more complex status of consecutive or even simultaneous injury, adaptation, and recovery [9,16,20,21].

## 3. Biochemical Mechanisms in IRI

**Figure 1:**
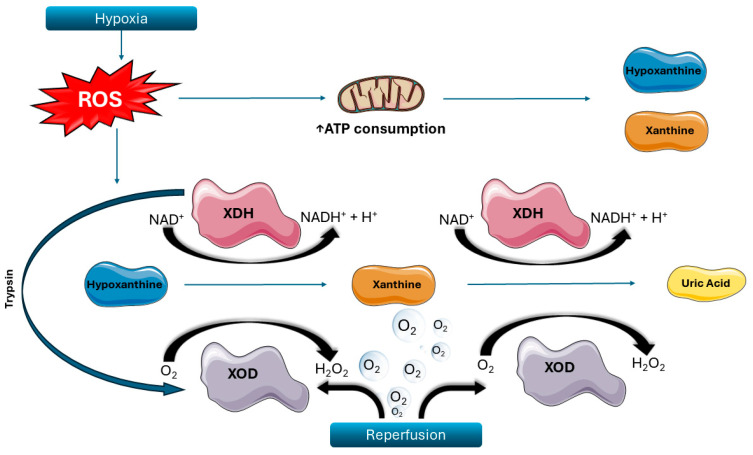
Biochemical mechanisms of oxidative stress during ischemia–reperfusion. The biochemical mechanisms underlying reperfusion injury involve the local generation of reactive oxygen species (ROS) and the transformation of xanthine dehydrogenase (XDH) into xanthine oxidase (XOD). This figure depicts the enzymatic processes and oxidative stress response within ischemic cells. During hypoxia, ROS levels rise, leading to the depletion of adenosine triphosphate (ATP) and the subsequent accumulation of hypoxanthine and xanthine. Under normoxic conditions, XDH utilizes nicotinamide adenine dinucleotide (NAD) to metabolize these substrates, producing reduced NAD and uric acid. In contrast, hypoxic stress promotes the conversion of XDH to XOD via irreversible proteolysis, primarily mediated by trypsin. Unlike XDH, XOD uses molecular oxygen as a cofactor, resulting in the formation of hydrogen peroxide (H_2_O_2_) upon reperfusion. Elevated intracellular H_2_O_2_ further amplifies ROS production, intensifying oxidative damage in the cell. Source: Reproduced from Archontakis-Barakakis, P.; Mavridis, T.; Chlorogiannis, D.D.; Barakakis, G.; Laou, E.; Sessler, D.I.; Gkiokas, G.; Chalkias, A. (2025) [8]. Intestinal oxygen utilisation and cellular adaptation during intestinal ischaemia-reperfusion injury. Clinical and translational medicine 2025, 15, e70136, © 2024 The Authors, licensed under CC BY 4.0 (https://creativecommons.org/licenses/by/4.0/, accessed on 30 July 2025). No changes were made. Available at: https://doi.org/10.1002/ctm2.70136 (accessed on 30 July 2025). ROS: reactive oxygen species; ATP: adenosine triphosphate; NAD: nicotinamide adenine dinucleotide; XDH: xanthine dehydrogenase; XOD: xanthine oxidase; H_2_O_2_: hydrogen peroxide.

Hydrogen peroxide and reactive oxygen species (ROS) are produced from the interaction between hydrogen oxide, oxygen, and other cellular contents [22,23,24,25,26,27,28,29,30,31,32,33,34,35,36,37,38,39,40,41]. After the onset of ischemia, adenosine triphosphate (ATP) is rapidly consumed [42], while the function of several cellular enzymes is profoundly altered. In hypoxic conditions, xanthine dehydrogenase (XDH), which under normal circumstances uses xanthine as a substrate and nicotinamide adenine dinucleotide (NAD) as a cofactor to produce uric acid, is modified via sulfhydryl oxidation or proteolysis to become xanthine oxidase (XOD). This new enzyme again metabolizes xanthine (or hypoxanthine),but instead utilizes oxygen as a cofactor to generate hydrogen peroxide [36,43]. The abundance of oxygen during the reperfusion phase accelerates XOD activity and, thus, the increase in intracellular hydrogen peroxide (Figure 1). The latter subsequently interacts with other intracellular compounds to produce other oxygen-based free radicals and ROS, damaging intracellular structures [25,26,27,44].

The second notable source of ROS is mitochondria. During ischemia, the oxygen flow through the mitochondrial electron transport chain is disrupted. This has an ROS-generating effect via different pathways. On the one hand, superoxide (O_2_^−^) molecules already embedded in the chain rapidly interact with water molecules to produce hydrogen peroxide. This is possible because the enzymes that typically oversee superoxide scavenging and detoxification, such as glutathione S-transferase and NADPH quinone oxidoreductase, go dormant. They become dormant when their respective substrates become depleted, as substrate availability is directly linked to ATP abundance [45,46]. On the other hand, the ability of the transporters embedded in mitochondrial membranes to confine metabolic waste is compromised when ATP levels are insufficient to support their function [47]. Furthermore, in hypoxic conditions, mitochondrial permeability transition pores (mPTP) develop quicker and remain open for longer periods. This facilitates the cytoplasmic release of hydrogen peroxide, ROS species, and other substances that are isolated within mitochondria under physiological conditions, such as mitochondrial matrix mitochondrial DNA and cytochrome C [47,48,49].

Moreover, the enzyme family of nicotinamide adenine dinucleotide phosphate oxidases (NOX) contributes to extracellular ROS production. These enzymes are activated by extracellular signals and alter the extracellular environment [47,50,51,52]. In normoxic conditions, two nitric oxide synthase (NOS) monomers combine to create the activated enzyme and generate nitric oxide (NO). Under IRI conditions, tetrahydrobiopterin, which functions as a cofactor, is oxidized to dihydrobiopterin, thus becoming unavailable, and the monomers end up operating independently to produce hydrogen peroxide [47,53].

The accumulation of ROS and hydrogen peroxide has two effects. First, they cause irreversible changes and functional compromises in a range of intracellular enzymes and protein structures, mainly by interacting with specific proteinic components (i.e., the cysteine, lysine, proline, arginine, and histidine amino acids) [54]. In addition, they impose a considerably more detrimental effect on DNA. Their interaction with the DNA sugar-phosphate backbone causes breaks to the sequence and their interaction with the nucleobases changes them chemically. The net effect is the destruction of the DNA structure and its ability to retain genetic information [55]. Strong chemotaxis is the second consequence of elevated hydrogen peroxide and ROS concentrations. Chemotaxis-induced neutrophil aggregation in the affected tissue is the most prominent immunological response during IRI [56].

## 4. The Hypoxia-Inducible Factor System

Cellular adjustment to hypoxia is mediated by a family of transcription factors that are collectively known as Hypoxia-Inducible Factors (HIFs). In a nutshell, and upon activation, they augment the transcription of certain gene sets and suppress others in order to preserve cellular metabolism and core cellular functions.

**Figure 2:**
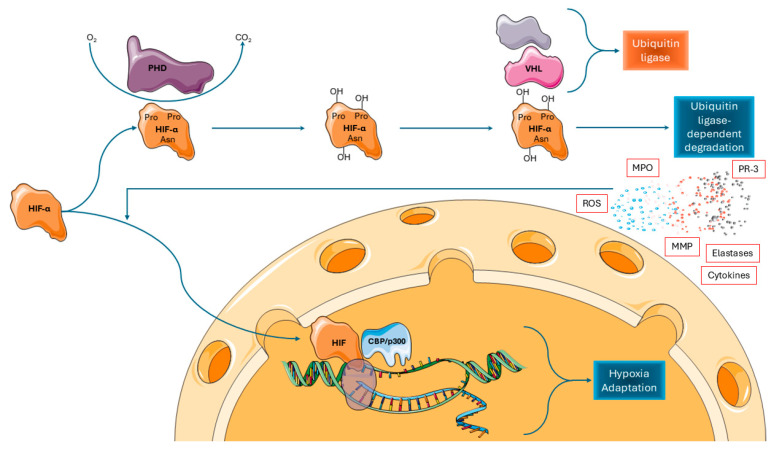
Oxygen-dependent regulation and activation of hypoxia-inducible factor-α (HIF-α). In the presence of normal oxygen levels (normoxia), HIF-α is rapidly degraded through the action of prolyl hydroxylase domain (PHD) enzymes. However, in the hypoxic intestinal environment, this degradation pathway is markedly inhibited, allowing HIF-α to accumulate and translocate into the nucleus. There, it interacts with the histone acetyltransferases CBP and p300, leading to the transcriptional activation of a range of target genes. Source: Reproduced from Archontakis-Barakakis, P.; Mavridis, T.; Chlorogiannis, D.D.; Barakakis, G.; Laou, E.; Sessler, D.I.; Gkiokas, G.; Chalkias, A. (2025) [8]. Intestinal oxygen utilisation and cellular adaptation during intestinal ischaemia-reperfusion injury. Clinical and translational medicine 2025, 15, e70136, © 2024 The Authors, licensed under CC BY 4.0 (https://creativecommons.org/licenses/by/4.0/, accessed on 30 July 2025). No changes were made. Available at: https://doi.org/10.1002/ctm2.70136, accessed on 30 July 2025. PHD: prolyl hydroxylase domain-containing proteins; HIF-α: hypoxia-inducible factor-α; VHL; von Hippel–Lindau tumor suppressor protein.

There are two HIF subunits, the alpha and the beta, that are separately produced and combine to form the active heterodimer structure of HIFs [57]. The ribosome production of the subunits and the formation of a complete HIF molecule occur at a relatively steady rate, but its catabolism varies widely according to oxygen availability; this represents the regulation step dictating the intracellular activity of HIFs. Under normoxic conditions, prolyl hydroxylase domain-containing (PHD) enzymes consume oxygen and hydroxylate the alpha subunit [58], thus creating a site for the Von-Hippel–Lindau (VHL) tumor suppressor protein to bind. VHL then poly-ubiquitinates the molecule, marking it for further degradation by proteasomes [59,60,61,62,63,64]. On the contrary, the hydroxylation process is massively reduced under hypoxic conditions. As a result, the alpha subunit’s rate of disintegration is slowed down and its concentration significantly rises. Now, the accessible alpha subunits can interact with the beta subunits more readily to create activated HIFs. The formed proteins translocate to the nucleus, bind to histone acetyl-transferases (CBP and p300), and form full activated transcription factors that can then alter the expression of gene sets [65,66,67,68,69]. This hypoxia sensing and responding mechanism has been described in all pertinent cell types in the GI tract, as described below [70,71] (Figure 2).

In addition to PHD, another enzyme family named Factors Inhibiting HIFs (FIHs) also play a role in regulating the HIF system. These proteins consume oxygen to hydroxylate an asparagine residue in the alpha subunit. This alteration decreases the affinity of HIFs with the p300 coactivator protein and, thus, does not allow the formation of active transcription factors in normoxic conditions [72].

It needs to be underlined that the HIF system is a critical cellular regulation and response mechanism that performs tasks beyond sensing oxygen levels [73]. This is demonstrated by the sheer number of chemical elements, proteins, and even other cells that influence its activity. First, hydrogen peroxide and other ROS species from all the aforementioned sources lead to an increase in intracellular concentrations of HIFs, although the precise biochemical mechanism is yet unknown [74]. Second, a similar effect has been described under carbon monoxide buildup, ammonia buildup [75], and under the influence of several cytokines, such as tumor necrosis factor-a (TNF-a) and interleukin-1b (IL-1b) [76,77,78], bacterial components such as lipopolysaccharides [79,80,81], and even whole bacteria. On the contrary, the increased intracellular presence of other compounds, such as nitric oxide, hydrogen sulfide, and carbon dioxide, has been associated with suppression of the HIF cascade [75]. Finally, multiple other crucial intracellular mechanisms, including the PI3K/AKT/mTOR pathway, the RAS/RAF/MEK/ERK pathway, and the IKK/NF-κB pathway, interact and affect the HIF system [82,83,84,85]. This particular connection is outside the purview of this review, and we direct the reader to other educational articles about the subject [8].

## 5. Intestinal Epithelial Cellular Mechanisms in IRI

The changes associated with HIF system activation include a wide range of cellular, tissue, and systemic manifestations. Particularly, the effect of HIFs causes intestinal epithelial cells (IECs) to change in both structure and function. The preservation of cellular connection to neighboring cells and the basement membrane is the most obvious structural alteration. Numerous transmembrane proteins, such as tight junctions, adherens junctions, and desmosomes, are present in the cell membrane of IECs and secure their attachment [86]. In an effort to strengthen inter-IEC attachment and maintain the stability of the epithelial lining and the epithelial barrier during hypoxia, the HIF system enhances the CLDN1 gene in IECs and boosts the synthesis and migration of Claudin-1 proteins towards the cellular membrane [87]. Apart from structural alterations, the HIF system enhances the MUC gene family and, thus, increases the production of mucins and the secretion of mucus. It has also been shown to increase the transcription of genes linked to other mucus-related proteins and other secretory functions of IECs, including the intestinal trefoil family factor (TFF) peptides, defensins, lysozyme, and secretory phospholipase 2 [88,89,90,91]. Overall, as a primary and rapid response, the mechanism has a favorable impact on intestinal lining integrity.

System activation does not, of course, guarantee complete protection from injury. If the cellular and tissue responses fail to compensate for the developing hypoxia, the epithelial lining of the affected GI tract loses its barrier function. The ability of IECs to generate the various proteins that create junctions and to direct them to the proper membrane placement is compromised, and the IECs’ attachment to surrounding structures is weakened [92,93,94]. When enough segments of the epithelial lining are destroyed, the damaged IECs become detached and disappear into the GI lumen due to the typically felt shearing forces of intestinal peristalsis. This phenomenon is even seen at the macroscale [18,95,96]. All of the luminal contents, with particular reference to intestinal bacteria, ingested toxins, and digestive enzymes released by the pancreas and liver, can enter the systemic circulation via infiltrating the gut and potentially the blood or lymphatic vessels [97].

In addition to HIF system exhaustion and failure, prolonged or strong and uncontrolled activation has also been linked with delirious effects, as evidenced by breakdown of the barrier faction, unmitigated bacterial infiltrations, and an uncontrolled inflammatory response [73].

## 6. Immune Cellular Mechanisms in IRI

The normal GI tract is populated by different types of immune cells, the primary being T lymphocytes. The normal T cell population of the GI tract belongs to two main subgroups. One consists of CD3-/CD8αβ^+^ cells that can be found in the lamina propria and among IECs; their primary function revolves around defending against bacteria, toxins, and other pathogens. The other group of T lymphocytes includes CD8αα^+^/CD8αα^−^ and CD4-/CD8αβ^−^ and has the primary task of immune response regulation to preserve epithelial homeostasis [98].

The first step of the immune response under IRI conditions is the release and circulation of different compounds that activate leucocytes. Most notable among them are different components of the IRI response, such as extracellularly available HIF-1, hydrogen peroxide, other ROS, cyclooxygenase-2, and poly(ADP-ribose) polymerase [99,100,101,102]. A similar immune activating effect is identified after the recognition and processing of the previously intraluminal material contaminating the affected tissue or even circulation (e.g., bacteria, digestion toxins, various exocrine enzymes) by leucocytes [103,104,105].

Except for these extracellular compounds, leucocyte activation is also mediated by their intracellular response mechanisms. The release of mitochondrial DNA into the cytoplasm provoked by hypoxic conditions, as described above, leads to its interaction with the endoplasmic Toll-like receptor-9 [106], a system usually tasked with bacterial and other pathogen recognition [107,108]. The NF-kB pathway is then activated and orchestrates the overproduction of cytokines and chemokines [106]. In addition, the intracellular buildup of hydrogen peroxide and other ROS species activates the nucleotide-binding domain and leucine rich repeat pyrin 3 (NLRP3) inflammasome. This subsequently activates the caspase 1 cascade, which also leads to chemokine and cytokine production and release (e.g., IL-1β and IL-18) [109,110,111].

After HIF activation, two main sets of changes have been described in intestinal T cells. The most extensively analyzed one, identified both in intestinal T cells and all other leukocyte subsets, is a shift in their metabolic profile. The SLC2A1 gene encoding the GLUT1 transporter and the genes encoding the various glycolysis enzymes (i.e., lactate dehydrogenase, pyruvate kinases, and phosphofructokinase 1) are upregulated by HIFs and their proteins gain larger concentrations and function intracellularly and on the cellular membrane. The net effect is a fundamental change from a primarily anaerobic to a primary anaerobic metabolism. In addition to altering the metabolism profile to adapt to the changing oxygen supply, this alteration represents a crucial activation cascade that causes cellular activation, heightened immunological response, and proliferation of the involved T cells [85,112,113]. The second and arguably less well understood effect set by HIF activation includes the recruitment of other immune cells from secondary lymphoid organs via cytokine generation, the proliferation of activated cells, and differentiation into memory cells. Based on the subset of T cells affected and the length of activation, the HIF system’s impact on these processes is considerably more complicated, with noticeable mixed consequences on the triggered cytokine production and the heightened inflammatory response, as evidenced by both beneficial and detrimental manifestations [113,114].

HIF pathway activation prolongs neutrophil survival and increases their phagocytic and cytotoxic function under IRI and hypoxic conditions. Again, the transition to a glycolysis-based metabolism is the metabolically significant step. This suppresses the well-known hypoxia-induced apoptosis, thus generating an increased survival effect in addition to functional activation of cells [115]. The composition of the neutrophil membrane receptors is also altered under HIF influence, with increased production of β2 integrins and formation of the full CD18 receptor. This facilitates the neutrophils’ extravasation after simpler endothelium adherence [116]. Their bactericidal and cytotoxic functions are also augmented. First, this is achieved through the increased generation of hydrogen peroxide and other ROS and their extracellular release [23,36,117,118]. Second, they are more capable of phagocytosing, degranulating, and releasing cytotoxic chemicals, including enzymes like metalloproteinases, myeloperoxidase, protease-3, and elastase, and they are more active in creating extracellular traps [40,119,120,121].

HIF-induced alterations play a crucial role in the development and resolution of gastrointestinal IRI, with dendritic cells and macrophages also involved. The metabolic transition to increased glycolysis has been documented for those immune cells as well. Regarding cell-specific alterations, the HIF cascade causes dendritic cells to produce and present more cellular receptors, including the CCR7 surface receptor, Toll-like receptor-2, and Toll-like receptor-6. This leads to greater phagocytosis and antigen presentation in addition to increased cell migration and maturation [122,123,124,125]. The profile of chemokines and cytokines produced by the dendritic cells is much less understood [85]. Macrophages also undergo a metabolic shift towards increased glycolysis, which results in polarization to their proinflammatory sub-type and augmentation of both their motility and phagocytosis capacity [126]. Substantial changes to their surface receptor profile, with increased concertation of CD40 and CD206 receptors, and to their chemokine and cytokine output, with increased excretion of IL-1β, TNF-α, and IL-6, have also been described [127,128,129,130].

## 7. Microcirculation and Tissue Changes in IRI

**Figure 3:**
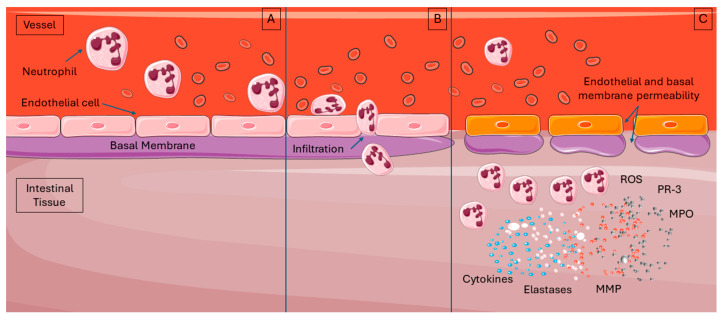
Neutrophil infiltration during the reperfusion phase of intestinal ischemia–reperfusion injury (IRI). (**A**). In the early stages of reperfusion, chemokines and cytokines mediate the recruitment of neutrophils to the intestinal tissue. (**B**). These neutrophils migrate into the affected area by disrupting the junctions between endothelial cells and traversing the endothelial basal membrane, resulting in markedly increased capillary permeability. (**C**). Once activated, neutrophils release various bioactive substances, including reactive oxygen species (ROS) and enzymes such as elastase, myeloperoxidase (MPO), protease-3 (PR-3), and matrix metalloproteinases. Source: Reproduced from Archontakis-Barakakis, P.; Mavridis, T.; Chlorogiannis, D.D.; Barakakis, G.; Laou, E.; Sessler, D.I.; Gkiokas, G.; Chalkias, A. (2025) [8]. Intestinal oxygen utilisation and cellular adaptation during intestinal ischaemia-reperfusion injury. Clinical and translational medicine 2025, 15, e70136, © 2024 The Authors, licensed under CC BY 4.0 (https://creativecommons.org/licenses/by/4.0/, accessed on 30 July 2025). No changes were made. Available at: https://doi.org/10.1002/ctm2.70136, accessed on 30 July 2025. ROS: reactive oxygen species; MPO: myeloperoxidase; PR-3: protease-3.

The process of neutrophil attraction and activation results in a steep increase in capillary permeability. Following chemoattraction to the affected tissue, the neutrophils start to adhere to the capillary endothelium via their interaction first with endothelial selectins, then with integrins, and finally, once stabilized, they migrate throughout the endothelial wall into the tissue [131,132]. This extravasation creates holes in the basement membrane between the endothelial cells and, thus, severely compromises barrier function, thus allowing other blood components, proteins, and cells to enter the tissue [132]. Intestinal tissue impacted by IRI is characterized microscopically by a high concentration of active neutrophils, whose presence also contributes to tissue hypoxia. These cells use any locally available oxygen for their immunologic functions, as previously described, a condition which contributes to existing hypoxia and causes even more damage accumulation [121,133]. These functions have an intended effect to suppress pathogens of course, but they also injure native tissue components (Figure 3).

In addition to capillary permeability changes, both the physical forces that are naturally applied to these vessels and the microcirculation contents change during IRI conditions. First, the various blood proteins which penetrate the impacted tissue as capillaries’ permeability rises cause the extracellular colloid pressure to increase and perivascular edema to form. This combination exerts an ever-increasing external pressure on the capillaries, the arterioles, and the venules, which can collapse, further contributing to ischemia [134]. Second, the hypoxic conditions and immune cell infiltration cause endothelial cell dysfunction and damage that manifests as endothelial edema and decreases the available circulation intravascular space [135]. Third, another phenomenon that compromises microcirculation includes the circulating leucocytes and specifically the activated neutrophils. These cells can initially adhere to the endothelium, as previously described, but then fail to extravasate and instead stagnate intravascularly, forming leucocyte aggregates. Lastly, the coagulation cascade is also triggered in hypoxic conditions. When the capillary wall breaks down, tissue factors are revealed and activate both the circulating platelets and the intrinsic and extrinsic coagulation pathways to form a complete clot. When all these factors come together, they further impair capillary flow and worsen hypoxia, resulting in recurrent cycles of IRI in the same damaged tissue [136,137].

**Figure 4:**
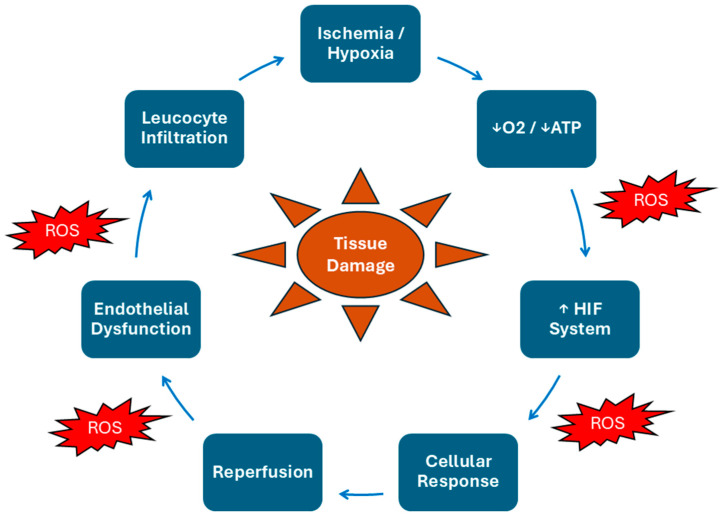
The ischemia–reperfusion vicious cycle. This schematic summarizes the self-perpetuating cycle of intestinal ischemia–reperfusion injury. Ischemia leads to oxygen and ATP depletion, triggering the HIF system and cellular stress. Reperfusion restores blood flow but causes a surge of ROS, which worsens tissue damage. ROS also promote endothelial dysfunction and leukocyte infiltration, further impairing perfusion and sustaining injury. The continuous interplay of hypoxia, ROS, and inflammation leads to progressive tissue damage and loss of epithelial integrity. ATP: Adenosine Triphosphate; HIF: Hypoxia-Inducible Factor; ROS: Reactive Oxygen Species.

The disruption of tissue structure under IRI conditions requires special attention. The extracellular environment and specifically the extracellular matrix of affected tissues are deteriorated by a family of zinc-dependent proteinases and extracellularly active enzymes known as metalloproteinases (MMPs) during IRI [138]. Under normal conditions, the GI tract’s leucocytes and IECs form and secrete the aforementioned factors locally at a low rate, demonstrating little activity [139,140]. However, during IRI, their production, secretion, and activity are significantly increased [141]. It has been demonstrated that the activation of the HIF system causes this rise only for particular metalloproteinases, primarily MMP-1 [142], MMP-15 [143], and MMP-17 [144]. However, the two systems seem to have multiple levels of interaction, as the activity of MMPs is also augmented by the presence of different types of ROS, which are also abundant during IRI conditions [145,146,147]. The net effect of this buildup is structural damage to the afflicted intestinal tissue and loss of the natural mechanical properties necessary for its function. The whole cycle of ischemia–reperfusion and the interplay of its different components are depicted schematically in Figure 4.

## 8. Clinical and Experimental Indicators for Diagnosing and Monitoring Intestinal Ischemia and Reperfusion

While the primary focus of this review is on the molecular and cellular mechanisms of IRI, highlighting the clinical and experimental parameters used to diagnose and monitor the progression of ischemia and reperfusion is critical. This section provides an overview of key diagnostic tools, laboratory indicators, and dynamic monitoring approaches that help define the stages of ischemia, assess the transition to irreversible damage, and characterize reperfusion dynamics [9].

### 8.1. Criteria for Defining the Ischemic Phase

There are no strict or universally accepted laboratory criteria expressed in numerical values to define the ischemic phase of IRI. Instead, ischemia is operationally characterized by a complete or near-complete reduction in intestinal blood flow, with experimental models typically inducing ≥90% occlusion of the superior mesenteric artery (SMA) to simulate ischemia. The duration of ischemia is another defining parameter, commonly ranging between 30 and 90 min, with 45 to 60 min being the most frequently employed timeframe in animal models, such as rats, dogs, and pigs, to induce reproducible injury while avoiding total necrosis (Table 1) [9,19]. In clinical practice, the diagnosis of intestinal ischemia does not rely on laboratory cut-offs, but rather on radiological indicators, such as the absence of bowel wall enhancement on CT angiography or direct intraoperative findings indicative of compromised perfusion.

### 8.2. Criteria for Irreversible Ischemia

Irreversible changes during intestinal ischemia occur when the duration and severity of hypoperfusion surpass the tissue’s compensatory capacity. Histologically and pathophysiologically, this is marked by near-complete ATP depletion, typically more than a 70% drop from baseline [39]. Concurrently, there is massive intracellular calcium influx, activating proteases and lipases, while mitochondrial permeability transition leads to a loss of membrane potential. This results in a loss of cell membrane integrity and the leakage of intracellular components. Tissue PO_2_, normally ~40–60 mmHg in the intestinal mucosa, drops below 10 mmHg during severe ischemia, and sustained PO_2_ values near 0–5 mmHg for over an hour are closely associated with irreversible injury [148]. Experimentally, complete occlusion of the mesenteric artery for ≥90–120 min reliably produces irreversible transmural necrosis [8]. Systemic indicators further reflect these changes; serum lactate levels of >4–6 mmol/L and a base deficit of >6–8 mmol/L are strongly correlated with irreversible intestinal injury and poor clinical outcomes. These parameters, though variable across models and clinical contexts, provide a quantitative framework to understand the transition from reversible to irreversible ischemic damage (Table 2).

### 8.3. Monitoring Intestinal Oxygenation During Ischemic Insult

Monitoring oxygenation is critical in the assessment of intestinal ischemia, yet conventional systemic measures such as pulse oximetry (SpO_2_) and arterial blood gases (PaO_2_) are insufficient and cannot detect local gut hypoxia properly. Instead, other techniques can be utilized to provide information regarding regional or tissue status. Tissue oxygen saturation (StO_2_), which reflects the balance between oxyhemoglobin and deoxyhemoglobin in the microvasculature (arterioles, capillaries, venules), can be measured via near-infrared spectroscopy (NIRS) and offers a practical albeit still experimental tool for intestinal ischemia monitoring. Studies suggest that StO_2_ levels below 60–70% in gastrointestinal tissues may indicate significant hypoxia [149,150]. Additionally, direct measurements of mucosal oxygenation, either directly via microelectrodes or tonometry, provide fairly accurate data, with mucosal PO_2_ values dropping below 10 mmHg indicating critical ischemia [151]. A third way to approximate tissue oxygenation is by calculating the oxy-/deoxyhemoglobin ratio that StO_2_ captures inherently; NIRS calculates StO_2_ by measuring the ratio of oxyhemoglobin to total hemoglobin in the microvascular bed (arteries, capillaries, venules). Clinically, combining StO_2_ monitoring with systemic biomarkers such as serum lactate (>4–6 mmol/L), base deficit (>6–8 mmol/L), and imaging modalities like CT angiography enhances diagnostic accuracy (Table 3). While bedside StO_2_ assessment is well established in trauma and critical care, its application in intestinal ischemia remains largely experimental. The proposed intraoperative monitoring strategies, including near-infrared spectroscopy for assessing intestinal wall oxygenation and mucosal tonometry for evaluating gastric or intestinal mucosal perfusion, are depicted in Table 4 and Table 5.

### 8.4. Diagnostic Indicators of Reperfusion Quality

The dynamics of reperfusion critically influence the path that will be followed, either towards tissue recovery or further injury. While no single diagnostic parameter can definitively predict trajectory, combining more than one parameter to monitor tissue oxygenation offers practical insights. A gradual, controlled increase in StO_2_ or mucosal PO_2_ is generally protective, whereas a rapid increase (typically a StO_2_ increase more than 20–30% within 1–2 min) suggests an oxidative burst and, thus, a higher reperfusion injury risk [149]. Similarly, a persistently elevated mucosal–arterial ΔPCO_2_ (PCO_2_ gap > 20 mmHg) after reperfusion indicates microvascular dysfunction, no-reflow phenomenon, and poor outcomes [151]. Other biochemical markers can be facilitated to measure oxidative stress. High malondialdehyde (MDA) levels (>2–3 µmol/L) are indicative of ROS damage and, thus, significant reperfusion injury [37]. Serum lactate dynamics also serve as a useful indirect marker, where a paradoxical increase of >2 mmol/L post-reperfusion suggests mitochondrial injury and ongoing anaerobic metabolism. Combining these indicators with Doppler flow assessments and clinical observations can help infer the ‘smoothness’ of reperfusion and anticipate potential injury (Table 6) [37,149].

## 9. Conclusions

The role of hypoxic conditions in the development of the changes identified during IRI is widely documented. The diagnosis of hypoxia and the coordination of all the chemical, subcellular, cellular, tissue, and even organ-level alterations reported over decades for IRI are primarily and critically dependent on the HIF system.

Beyond these mechanisms, clinical monitoring tools such as StO_2_ via NIRS, mucosal PO, and biomarkers like lactate may be useful for assessing ischemia severity and reperfusion dynamics. Improved diagnostic monitoring could enhance early detection and intervention, preserving intestinal viability and improving outcomes.

Future research should focus on validating diagnostic thresholds and integrating molecular insights into clinical practice to better manage intestinal IRI.

## Figures and Tables

**Table 1:** Supporting numerical lab indicators for intestinal ischemia.

Parameter	Typical Indicator in Ischemia
Lactate	>2 mmol/L (severe cases often >4 mmol/L)
pH/Base deficit	pH < 7.35, base deficit > 4 mmol/L
Intestinal fatty acid-binding protein (I-FABP)	Elevated compared to baseline—no single cutoff; used as an early marker of mucosal damage
D-lactate	Elevated; typical serum levels rise above 0.25 mmol/L, but ranges vary
Cytokines (e.g., TNF-α, IL-6)	Elevated; used in research, no diagnostic cutoff

Although these biomarkers can support clinical suspicion, they are not specific nor sufficient to define the ischemic phase of ischemia–reperfusion injury (IRI) on their own. Cutoff values, where available, are indicative and may vary across studies and clinical settings. I-FABP: Intestinal Fatty Acid-Binding Protein; TNF-α: Tumor Necrosis Factor-alpha; IL-6: Interleukin-6; IRI: Ischemia–Reperfusion Injury; SMA: Superior Mesenteric Artery.

**Table 2:** Supporting numerical lab indicators for irreversible intestinal ischemia.

Parameter	Approximate Irreversible Range
SMA occlusion time	≥90–120 min (animal models)
Tissue ATP	≤10–30% of baseline (~0.3–0.5 µmol/g)
Mucosal PO_2_	~0–5 mmHg for >60 min
Serum lactate (clinical)	>4–6 mmol/L → high risk of transmural necrosis
Histopathology	Full-thickness (transmural) necrosis, loss of mucosal architecture

This table summarizes experimental and clinical parameters that correlate with irreversible intestinal ischemia. Values reflect thresholds beyond which tissue damage progresses to necrosis and loss of function. While these indicators guide interpretation, clinical variability and experimental conditions may influence thresholds. SMA: Superior Mesenteric Artery; ATP: Adenosine Triphosphate; PO_2_: Partial Pressure of Oxygen.

**Table 3:** Usefulness of different parameters for diagnosing intestinal ischemia.

Parameter	Approximate Irreversible Range
Systemic SpO_2_	Not useful for local ischemia
Hb concentration	Not useful by itself
Lactate/base deficit	Good general markers of anaerobic metabolism
StO_2_ (NIRS)	Most practical noninvasive method to estimate local tissue oxygenation
Mucosal PO_2_/PCO_2_	Excellent but more invasive, used in research/ICU
Imaging	CT angiography

This table summarizes the relative utility of systemic, regional, and tissue-specific parameters in identifying intestinal ischemia and assessing its severity. While some markers are general indicators of metabolic distress, others provide more direct insights into local tissue perfusion and oxygenation. SpO_2_: Oxygen saturation (measured by pulse oximetry); Hb: Hemoglobin; StO_2_: Tissue Oxygen Saturation; NIRS: Near-Infrared Spectroscopy; PO_2_: Partial Pressure of Oxygen; PCO_2_: Partial Pressure of Carbon Dioxide; ICU: Intensive Care Unit; CT: Computed Tomography.

**Table 4:** Proposed protocol of intraoperative NIRS for gut StO_2_.

Intraoperative NIRS for Gut StO_2_
**Purpose**
To assess local tissue oxygen saturation (StO_2_) in the intestinal wall during surgery for suspected ischemia (e.g., bowel resection, mesenteric thrombectomy).
**Equipment**
**Near-Infrared Spectroscopy (NIRS)** monitor, e.g., INVOS™, NIRO™, or equivalent.
Sterile adhesive sensor/probe for intraoperative use.
Optional: Doppler probe to confirm perfusion.
**Protocol Steps**
**1. Patient prep**
Standard anesthesia, maintain normoxia (SpO_2_ > 95%), normotension.
Baseline arterial blood gas, lactate, base deficit.
**2. Probe placement**
Sterilize probe surface if reusable.
Gently place the NIRS probe directly onto the serosal surface of the bowel loop in question.
Avoid compressing the bowel wall to prevent false readings.
**3. Measurement**
Wait 30–60 s for signal stabilization.
Record baseline StO_2_.
Take readings every 5–10 min, or continuously if device allows.
Repeat after interventions: e.g., SMA release, anastomosis.
**4. Interpretation**
Normal StO_2_ for gut wall: ~70–80%
StO_2_ < 60–70% → suspicious for inadequate perfusion.
Persistent StO_2_ < 50–60% despite revascularization → consider nonviable segment.
**5. Documentation**
Note probe site, time, and conditions.
Correlate with clinical judgement (color, peristalsis, Doppler flow)

This table outlines a step-by-step protocol for the intraoperative use of NIRS to monitor local StO_2_ in the intestinal wall. The protocol includes equipment requirements, procedural steps, interpretation thresholds for StO_2_ values, and documentation guidelines. Modified from reference [149]. SpO_2_: Oxygen saturation (measured by pulse oximetry); StO_2_: Tissue Oxygen Saturation; NIRS: Near-Infrared Spectroscopy; SMA: Superior Mesenteric Artery.

**Table 5:** Proposed protocol of mucosal tonometry for gastric or intestinal mucosal oxygenation.

Mucosal Tonometry for Gastric or Intestinal Mucosal Oxygenation
**Purpose**
To indirectly assess mucosal hypoxia by measuring local mucosal PCO_2_ or mucosal pH (pHi) → increased mucosal PCO_2_ means hypoperfusion.
**Equipment**
Gastric or intestinal tonometer catheter (e.g., TRIP tonometer or balloon tonometry).
Standard blood gas analyzer.
**Protocol Steps**
**1. Patient prep**
Insert tonometry catheter into stomach or directly into bowel lumen if intraoperative.
Fill balloon with air or saline according to device instructions.
**2. Equilibration**
**3. Sampling**
Aspirate sample gas from balloon.
Measure PCO_2_ using standard ABG machine.
Simultaneously measure arterial blood PCO_2_.
**4. Calculations**
Calculate mucosal–arterial CO_2_ gap (ΔPCO_2_): ΔPCO_2_ = PCO_2_(mucosa)—PCO_2_(arterial)
Calculate gastric pHi: pHi = 6.1 + log([HCO_3_^−^]/(PCO_2_ mucosal × 0.03))
**5. Interpretation**
Normal mucosal–arterial CO_2_ gap: <15 mmHg.
ΔPCO_2_ > 20 mmHg or pHi < 7.3 → significant mucosal ischemia.
Persistent elevation → high risk of irreversible damage.

This table presents a structured protocol for assessing gastrointestinal mucosal oxygenation using mucosal tonometry. The protocol outlines required equipment, procedural steps, calculation formulas, interpretation thresholds, and clinical implications for detecting inadequate perfusion. Μodified from reference [151]. PCO_2_: Partial Pressure of Carbon Dioxide; pHi: Intramucosal pH; ΔPCO_2_: Mucosal–Arterial Carbon Dioxide Gap; ABG: Arterial Blood Gas; [HCO_3_^−^]: Bicarbonate Concentration.

**Table 6:** Criteria to determine the dynamics of reperfusion.

Parameter	Controlled (Smooth) Reperfusion	Risk of Reperfusion Injury
StO_2_ (NIRS)	Gradual rise to 60–80% over 5–10 min	Sudden spike > 20–30% in 1–2 min
Mucosal–arterial CO_2_ gap	Normalizes to <15 mmHg	Remains > 20 mmHg
Plasma MDA	Stable, low	Peaks > 2–3 µmol/L
XOD activity	Mild increase	High levels
MPO	Mild increase	High levels
Serum lactate	Steady fall	Increased Paradoxical rebound
Doppler flow Microvascular flow	Laminar, adequate	Turbulent flow Microvascular no-reflow pattern

This table provides parameters that can be monitored to effectively diagnose reperfusion injury. Values are indicative of smooth versus abnormal reperfusion (IRI). StO_2_: Tissue Oxygen Saturation; NIRS: Near-Infrared Spectroscopy; MDA: Malondialdehyde; XOD, xanthine oxidase; MPO, myeloperoxidase.

## Data Availability

Our data is derived from public domain resources. All data source material that supports the findings of this study is available on Medline.

## References

[1] Kalogeris T., Baines C.P., Krenz M., Korthuis R.J. (2016). Ischemia/Reperfusion. Compr. Physiol..

[2] Zhao W., Gan X., Su G., Wanling G., Li S., Hei Z., Yang C., Wang H. (2014). The interaction between oxidative stress and mast cell activation plays a role in acute lung injuries induced by intestinal ischemia-reperfusion. J. Surg. Res..

[3] Deitch E.A., Xu D., Kaise V.L. (2006). Role of the gut in the development of injury- and shock induced SIRS and MODS: The gut-lymph hypothesis, a review. Front. Biosci..

[4] Huang C.Y., Hsiao J.K., Lu Y.Z., Lee T.C., Yu L.C. (2011). Anti-apoptotic PI3K/Akt signaling by sodium/glucose transporter 1 reduces epithelial barrier damage and bacterial translocation in intestinal ischemia. Lab. Investig..

[5] Puleo F., Arvanitakis M., Van Gossum A., Preiser J.C. (2011). Gut failure in the ICU. Semin. Respir. Crit. Care Med..

[6] Swank G.M., Deitch E.A. (1996). Role of the gut in multiple organ failure: Bacterial translocation and permeability changes. World J. Surg..

[7] Yasuhara H. (2005). Acute mesenteric ischemia: The challenge of gastroenterology. Surg. Today.

[8] Archontakis-Barakakis P., Mavridis T., Chlorogiannis D.D., Barakakis G., Laou E., Sessler D.I., Gkiokas G., Chalkias A. (2025). Intestinal oxygen utilisation and cellular adaptation during intestinal ischaemia-reperfusion injury. Clin. Transl. Med..

[9] Mallick I.H., Yang W., Winslet M.C., Seifalian A.M. (2004). Ischemia-reperfusion injury of the intestine and protective strategies against injury. Dig. Dis. Sci..

[10] Sastry P., Hardman G., Page A., Parker R., Goddard M., Large S., Jenkins D.P. (2014). Mesenteric ischaemia following cardiac surgery: The influence of intraoperative perfusion parameters. Interact. Cardiovasc. Thorac. Surg..

[11] Corcos O., Nuzzo A. (2013). Gastro-intestinal vascular emergencies. Best. Pract. Res. Clin. Gastroenterol..

[12] Fishman J.E., Sheth S.U., Levy G., Alli V., Lu Q., Xu D., Qin Y., Qin X., Deitch E.A. (2014). Intraluminal nonbacterial intestinal components control gut and lung injury after trauma hemorrhagic shock. Ann. Surg..

[13] Brandt L.J., Feuerstadt P., Longstreth G.F., Boley S.J., American College of Gastroenterology (2015). ACG clinical guideline: Epidemiology, risk factors, patterns of presentation, diagnosis, and management of colon ischemia (CI). Am. J. Gastroenterol..

[14] Acosta S. (2010). Epidemiology of mesenteric vascular disease: Clinical implications. Semin. Vasc. Surg..

[15] Darien B.J., Sims P.A., Stone W.C., Schilly D.R., Dubielzig R.R., Albrecht R.M. (1993). Ischemia/reperfusion injury of the ascending colon in ponies: A correlative study utilizing microvascular histopathology and corrosion casting. Scanning Microsc..

[16] Granger D.N., Korthuis R.J. (1995). Physiologic mechanisms of postischemic tissue injury. Annu. Rev. Physiol..

[17] Blikslager A.T. (2008). Life in the gut without oxygen: Adaptive mechanisms and inflammatory bowel disease. Gastroenterology.

[18] Chiu C.J., McArdle A.H., Brown R., Scott H.J., Gurd F.N. (1970). Intestinal mucosal lesion in low-flow states. I. A morphological, hemodynamic, and metabolic reappraisal. Arch. Surg..

[19] Derikx J.P., Matthijsen R.A., de Bruine A.P., van Bijnen A.A., Heineman E., van Dam R.M., Dejong C.H., Buurman W.A. (2008). Rapid reversal of human intestinal ischemia-reperfusion induced damage by shedding of injured enterocytes and reepithelialisation. PLoS ONE.

[20] Hernandez G., Bruhn A., Luengo C., Regueira T., Kattan E., Fuentealba A., Florez J., Castro R., Aquevedo A., Pairumani R. (2013). Effects of dobutamine on systemic, regional and microcirculatory perfusion parameters in septic shock: A randomized, placebo-controlled, double-blind, crossover study. Intensive Care Med..

[21] Sinaasappel M., van Iterson M., Ince C. (1999). Microvascular oxygen pressure in the pig intestine during haemorrhagic shock and resuscitation. J. Physiol..

[22] Boros M., Takaichi S., Hatanaka K. (1995). Ischemic time-dependent microvascular changes and reperfusion injury in the rat small intestine. J. Surg. Res..

[23] Deshmukh D.R., Mirochnitchenko O., Ghole V.S., Agnese D., Shah P.C., Reddell M., Brolin R.E., Inouye M. (1997). Intestinal ischemia and reperfusion injury in transgenic mice overexpressing copper-zinc superoxide dismutase. Am. J. Physiol..

[24] Granger D.N., Benoit J.N., Suzuki M., Grisham M.B. (1989). Leukocyte adherence to venular endothelium during ischemia-reperfusion. Am. J. Physiol..

[25] Granger D.N., Hollwarth M.E., Parks D.A. (1986). Ischemia-reperfusion injury: Role of oxygen-derived free radicals. Acta Physiol. Scand. Suppl..

[26] Granger D.N., Rutili G., McCord J.M. (1981). Superoxide radicals in feline intestinal ischemia. Gastroenterology.

[27] Grisham M.B., Hernandez L.A., Granger D.N. (1986). Xanthine oxidase and neutrophil infiltration in intestinal ischemia. Am. J. Physiol..

[28] Grum C.M., Gross T.J., Mody C.H., Sitrin R.G. (1990). Expression of xanthine oxidase activity by murine leukocytes. J. Lab. Clin. Med..

[29] Haglind E., Haglund U., Lundgren O., Stenberg B. (1985). Mucosal lesions of the small intestine after intestinal vascular obstruction in the rat. Acta Chir. Scand..

[30] Hernandez L.A., Grisham M.B., Granger D.N. (1987). A role for iron in oxidant-mediated ischemic injury to intestinal microvasculature. Am. J. Physiol..

[31] Kubes P., Hunter J., Granger D.N. (1992). Ischemia/reperfusion-induced feline intestinal dysfunction: Importance of granulocyte recruitment. Gastroenterology.

[32] Kubes P., Hunter J., Granger D.N. (1991). Effects of cyclosporin A and FK506 on ischemia/reperfusion-induced neutrophil infiltration in the cat. Dig. Dis. Sci..

[33] Kurtel H., Tso P., Granger D.N. (1992). Granulocyte accumulation in postischemic intestine: Role of leukocyte adhesion glycoprotein CD11/CD18. Am. J. Physiol..

[34] Nilsson U.A., Aberg J., Aneman A., Lundgren O. (1993). Feline intestinal ischemia and reperfusion: Relation between radical formation and tissue damage. Eur. Surg. Res..

[35] Nilsson U.A., Lundgren O., Haglind E., Bylund-Fellenius A.C. (1989). Radical production during in vivo intestinal ischemia and reperfusion in the cat. Am. J. Physiol..

[36] Nilsson U.A., Schoenberg M.H., Aneman A., Poch B., Magadum S., Beger H.G., Lundgren O. (1994). Free radicals and pathogenesis during ischemia and reperfusion of the cat small intestine. Gastroenterology.

[37] Parks D.A., Bulkley G.B., Granger D.N., Hamilton S.R., McCord J.M. (1982). Ischemic injury in the cat small intestine: Role of superoxide radicals. Gastroenterology.

[38] Parks D.A., Granger D.N. (1986). Contributions of ischemia and reperfusion to mucosal lesion formation. Am. J. Physiol..

[39] Parks D.A., Granger D.N. (1983). Ischemia-induced vascular changes: Role of xanthine oxidase and hydroxyl radicals. Am. J. Physiol..

[40] Schoenberg M.H., Poch B., Younes M., Schwarz A., Baczako K., Lundberg C., Haglund U., Beger H.G. (1991). Involvement of neutrophils in postischaemic damage to the small intestine. Gut.

[41] Suzuki M., Grisham M.B., Granger D.N. (1991). Leukocyte-endothelial cell adhesive interactions: Role of xanthine oxidase-derived oxidants. J. Leukoc. Biol..

[42] Chaudry I.H. (1990). Use of ATP following shock and ischemia. Ann. N. Y. Acad. Sci..

[43] Chung H.Y., Baek B.S., Song S.H., Kim M.S., Huh J.I., Shim K.H., Kim K.W., Lee K.H. (1997). Xanthine dehydrogenase/xanthine oxidase and oxidative stress. Age.

[44] Granger D.N., McCord J.M., Parks D.A., Hollwarth M.E. (1986). Xanthine oxidase inhibitors attenuate ischemia-induced vascular permeability changes in the cat intestine. Gastroenterology.

[45] Hayes J.D., Flanagan J.U., Jowsey I.R. (2005). Glutathione transferases. Annu. Rev. Pharmacol. Toxicol..

[46] Jaiswal A.K. (2000). Characterization and partial purification of microsomal NAD(P)H:quinone oxidoreductases. Arch. Biochem. Biophys..

[47] Granger D.N., Kvietys P.R. (2015). Reperfusion injury and reactive oxygen species: The evolution of a concept. Redox Biol..

[48] Tissier R., Chenoune M., Pons S., Zini R., Darbera L., Lidouren F., Ghaleh B., Berdeaux A., Morin D. (2013). Mild hypothermia reduces per-ischemic reactive oxygen species production and preserves mitochondrial respiratory complexes. Resuscitation.

[49] Garcia N., Chavez E. (2007). Mitochondrial DNA fragments released through the permeability transition pore correspond to specific gene size. Life Sci..

[50] Kuwano Y., Tominaga K., Kawahara T., Sasaki H., Takeo K., Nishida K., Masuda K., Kawai T., Teshima-Kondo S., Rokutan K. (2008). Tumor necrosis factor alpha activates transcription of the NADPH oxidase organizer 1 (NOXO1) gene and upregulates superoxide production in colon epithelial cells. Free Radic. Biol. Med..

[51] El Hassani R.A., Benfares N., Caillou B., Talbot M., Sabourin J.C., Belotte V., Morand S., Gnidehou S., Agnandji D., Ohayon R. (2005). Dual oxidase2 is expressed all along the digestive tract. Am. J. Physiol. Gastrointest. Liver Physiol..

[52] Ha E.M., Oh C.T., Bae Y.S., Lee W.J. (2005). A direct role for dual oxidase in Drosophila gut immunity. Science.

[53] Kubes P., McCafferty D.M. (2000). Nitric oxide and intestinal inflammation. Am. J. Med..

[54] Dickinson B.C., Chang C.J. (2011). Chemistry and biology of reactive oxygen species in signaling or stress responses. Nat. Chem. Biol..

[55] Imlay J.A. (2013). The molecular mechanisms and physiological consequences of oxidative stress: Lessons from a model bacterium. Nat. Rev. Microbiol..

[56] Wang M., Zhang J., Gong N. (2022). Role of the PI3K/Akt signaling pathway in liver ischemia reperfusion injury: A narrative review. Ann. Palliat. Med..

[57] Wang F.S., Wang C.J., Chen Y.J., Chang P.R., Huang Y.T., Sun Y.C., Huang H.C., Yang Y.J., Yang K.D. (2004). Ras induction of superoxide activates ERK-dependent angiogenic transcription factor HIF-1alpha and VEGF-A expression in shock wave-stimulated osteoblasts. J. Biol. Chem..

[58] Fiorini G., Schofield C.J. (2024). Biochemistry of the hypoxia-inducible factor hydroxylases. Curr. Opin. Chem. Biol..

[59] Singhal R., Shah Y.M. (2020). Oxygen battle in the gut: Hypoxia and hypoxia-inducible factors in metabolic and inflammatory responses in the intestine. J. Biol. Chem..

[60] Heir P., Ohh M. (2016). Hydroxylation-Dependent Interaction of Substrates to the Von Hippel-Lindau Tumor Suppressor Protein (VHL). Methods Mol. Biol..

[61] Ohh M., Park C.W., Ivan M., Hoffman M.A., Kim T.Y., Huang L.E., Pavletich N., Chau V., Kaelin W.G. (2000). Ubiquitination of hypoxia-inducible factor requires direct binding to the beta-domain of the von Hippel-Lindau protein. Nat. Cell Biol..

[62] Maxwell P.H., Wiesener M.S., Chang G.W., Clifford S.C., Vaux E.C., Cockman M.E., Wykoff C.C., Pugh C.W., Maher E.R., Ratcliffe P.J. (1999). The tumour suppressor protein VHL targets hypoxia-inducible factors for oxygen-dependent proteolysis. Nature.

[63] Jewell U.R., Kvietikova I., Scheid A., Bauer C., Wenger R.H., Gassmann M. (2001). Induction of HIF-1alpha in response to hypoxia is instantaneous. FASEB J..

[64] Watts E.R., Walmsley S.R. (2019). Inflammation and Hypoxia: HIF and PHD Isoform Selectivity. Trends Mol. Med..

[65] Lando D., Peet D.J., Whelan D.A., Gorman J.J., Whitelaw M.L. (2002). Asparagine hydroxylation of the HIF transactivation domain a hypoxic switch. Science.

[66] Wenger R.H., Stiehl D.P., Camenisch G. (2005). Integration of oxygen signaling at the consensus HRE. Sci. STKE Signal Transduct. Knowl. Environ..

[67] Smythies J.A., Sun M., Masson N., Salama R., Simpson P.D., Murray E., Neumann V., Cockman M.E., Choudhry H., Ratcliffe P.J. (2019). Inherent DNA-binding specificities of the HIF-1alpha and HIF-2alpha transcription factors in chromatin. EMBO Rep..

[68] Kaelin W.G., Ratcliffe P.J. (2008). Oxygen sensing by metazoans: The central role of the HIF hydroxylase pathway. Mol. Cell.

[69] Taylor C.T., Scholz C.C. (2022). The effect of HIF on metabolism and immunity. Nat. Rev. Nephrol..

[70] Ramakrishnan S.K., Shah Y.M. (2016). Role of Intestinal HIF-2alpha in Health and Disease. Annu. Rev. Physiol..

[71] Sun L., Li T., Tang H., Yu K., Ma Y., Yu M., Qiu Y., Xu P., Xiao W., Yang H. (2019). Intestinal Epithelial Cells-Derived Hypoxia-Inducible Factor-1alpha Is Essential for the Homeostasis of Intestinal Intraepithelial Lymphocytes. Front. Immunol..

[72] Volkova Y.L., Pickel C., Jucht A.E., Wenger R.H., Scholz C.C. (2022). The Asparagine Hydroxylase FIH: A Unique Oxygen Sensor. Antioxid. Redox Signal..

[73] Feinman R., Deitch E.A., Watkins A.C., Abungu B., Colorado I., Kannan K.B., Sheth S.U., Caputo F.J., Lu Q., Ramanathan M. (2010). HIF-1 mediates pathogenic inflammatory responses to intestinal ischemia-reperfusion injury. Am. J. Physiol. Gastrointest. Liver Physiol..

[74] Martinez-Reyes I., Diebold L.P., Kong H., Schieber M., Huang H., Hensley C.T., Mehta M.M., Wang T., Santos J.H., Woychik R. (2016). TCA Cycle and Mitochondrial Membrane Potential Are Necessary for Diverse Biological Functions. Mol. Cell.

[75] Kalantar-Zadeh K., Berean K.J., Burgell R.E., Muir J.G., Gibson P.R. (2019). Intestinal gases: Influence on gut disorders and the role of dietary manipulations. Nat. Rev. Gastroenterol. Hepatol..

[76] Nizet V., Johnson R.S. (2009). Interdependence of hypoxic and innate immune responses. Nat. Rev. Immunol..

[77] Hellwig-Burgel T., Rutkowski K., Metzen E., Fandrey J., Jelkmann W. (1999). Interleukin-1beta and tumor necrosis factor-alpha stimulate DNA binding of hypoxia-inducible factor-1. Blood.

[78] Scharte M., Han X., Bertges D.J., Fink M.P., Delude R.L. (2003). Cytokines induce HIF-1 DNA binding and the expression of HIF-1-dependent genes in cultured rat enterocytes. Am. J. Physiol. Gastrointest. Liver Physiol..

[79] Koury J., Deitch E.A., Homma H., Abungu B., Gangurde P., Condon M.R., Lu Q., Xu D.Z., Feinman R. (2004). Persistent HIF-1alpha activation in gut ischemia/reperfusion injury: Potential role of bacteria and lipopolysaccharide. Shock.

[80] Huang L.E., Gu J., Schau M., Bunn H.F. (1998). Regulation of hypoxia-inducible factor 1alpha is mediated by an O_2_-dependent degradation domain via the ubiquitin-proteasome pathway. Proc. Natl. Acad. Sci. USA.

[81] Scharte M., Han X., Uchiyama T., Tawadrous Z., Delude R.L., Fink M.P. (2006). LPS increases hepatic HIF-1alpha protein and expression of the HIF-1-dependent gene aldolase A in rats. J. Surg. Res..

[82] Niu G., Briggs J., Deng J., Ma Y., Lee H., Kortylewski M., Kujawski M., Kay H., Cress W.D., Jove R. (2008). Signal transducer and activator of transcription 3 is required for hypoxia-inducible factor-1alpha RNA expression in both tumor cells and tumor-associated myeloid cells. Mol. Cancer Res. MCR.

[83] D’Hulst G., Soro-Arnaiz I., Masschelein E., Veys K., Fitzgerald G., Smeuninx B., Kim S., Deldicque L., Blaauw B., Carmeliet P. (2020). PHD1 controls muscle mTORC1 in a hydroxylation-independent manner by stabilizing leucyl tRNA synthetase. Nat. Commun..

[84] Masoud G.N., Li W. (2015). HIF-1alpha pathway: Role, regulation and intervention for cancer therapy. Acta Pharm. Sin. B.

[85] McGettrick A.F., O’Neill L.A.J. (2020). The Role of HIF in Immunity and Inflammation. Cell Metab..

[86] Laukoetter M.G., Bruewer M., Nusrat A. (2006). Regulation of the intestinal epithelial barrier by the apical junctional complex. Curr. Opin. Gastroenterol..

[87] Saeedi B.J., Kao D.J., Kitzenberg D.A., Dobrinskikh E., Schwisow K.D., Masterson J.C., Kendrick A.A., Kelly C.J., Bayless A.J., Kominsky D.J. (2015). HIF-dependent regulation of claudin-1 is central to intestinal epithelial tight junction integrity. Mol. Biol. Cell.

[88] Birchenough G.M., Johansson M.E., Gustafsson J.K., Bergstrom J.H., Hansson G.C. (2015). New developments in goblet cell mucus secretion and function. Mucosal Immunol..

[89] Bin Hafeez A., Jiang X., Bergen P.J., Zhu Y. (2021). Antimicrobial Peptides: An Update on Classifications and Databases. Int. J. Mol. Sci..

[90] Louis N.A., Hamilton K.E., Canny G., Shekels L.L., Ho S.B., Colgan S.P. (2006). Selective induction of mucin-3 by hypoxia in intestinal epithelia. J. Cell. Biochem..

[91] Krzywinska E., Stockmann C. (2018). Hypoxia, Metabolism and Immune Cell Function. Biomedicines.

[92] Kistler E.B., Alsaigh T., Chang M., Schmid-Schonbein G.W. (2012). Impaired small-bowel barrier integrity in the presence of lumenal pancreatic digestive enzymes leads to circulatory shock. Shock.

[93] Chang M., Alsaigh T., Kistler E.B., Schmid-Schonbein G.W. (2012). Breakdown of mucin as barrier to digestive enzymes in the ischemic rat small intestine. PLoS ONE.

[94] Alsaigh T., Chang M., Richter M., Mazor R., Kistler E.B. (2015). In vivo analysis of intestinal permeability following hemorrhagic shock. World J. Crit. Care Med..

[95] Blikslager A.T., Roberts M.C., Rhoads J.M., Argenzio R.A. (1997). Is reperfusion injury an important cause of mucosal damage after porcine intestinal ischemia?. Surgery.

[96] Park P.O., Haglund U., Bulkley G.B., Falt K. (1990). The sequence of development of intestinal tissue injury after strangulation ischemia and reperfusion. Surgery.

[97] DeLano F.A., Hoyt D.B., Schmid-Schonbein G.W. (2013). Pancreatic digestive enzyme blockade in the intestine increases survival after experimental shock. Sci. Transl. Med..

[98] Ma H., Tao W., Zhu S. (2019). T lymphocytes in the intestinal mucosa: Defense and tolerance. Cell Mol. Immunol..

[99] Michalsky M.P., Deitch E.A., Ding J., Lu Q., Huang Q. (1997). Interleukin-6 and tumor necrosis factor production in an enterocyte cell model (Caco-2) during exposure to Escherichia coli. Shock.

[100] Yeh K.Y., Yeh M., Glass J., Granger D.N. (2000). Rapid activation of NF-kappaB and AP-1 and target gene expression in postischemic rat intestine. Gastroenterology.

[101] Hierholzer C., Harbrecht B.G., Billiar T.R., Tweardy D.J. (2001). Hypoxia-inducible factor-1 activation and cyclo-oxygenase-2 induction are early reperfusion-independent inflammatory events in hemorrhagic shock. Arch. Orthop. Trauma. Surg..

[102] Liaudet L., Szabo A., Soriano F.G., Zingarelli B., Szabo C., Salzman A.L. (2000). Poly (ADP-ribose) synthetase mediates intestinal mucosal barrier dysfunction after mesenteric ischemia. Shock.

[103] Grootjans J., Lenaerts K., Derikx J.P., Matthijsen R.A., de Bruine A.P., van Bijnen A.A., van Dam R.M., Dejong C.H., Buurman W.A. (2010). Human intestinal ischemia-reperfusion-induced inflammation characterized: Experiences from a new translational model. Am. J. Pathol..

[104] Louis K., Netea M.G., Carrer D.P., Kotsaki A., Mylona V., Pistiki A., Savva A., Roditis K., Alexis A., Van der Meer J.W. (2013). Bacterial translocation in an experimental model of multiple organ dysfunctions. J. Surg. Res..

[105] Podolsky D.K. (1999). Mucosal immunity and inflammation. V. Innate mechanisms of mucosal defense and repair: The best offense is a good defense. Am. J. Physiol..

[106] Riley J.S., Tait S.W. (2020). Mitochondrial DNA in inflammation and immunity. EMBO Rep..

[107] Hemmi H., Takeuchi O., Kawai T., Kaisho T., Sato S., Sanjo H., Matsumoto M., Hoshino K., Wagner H., Takeda K. (2000). A Toll-like receptor recognizes bacterial DNA. Nature.

[108] Rose W.A., Sakamoto K., Leifer C.A. (2012). TLR9 is important for protection against intestinal damage and for intestinal repair. Sci. Rep..

[109] Liu C.Y., Cham C.M., Chang E.B. (2021). Epithelial wound healing in inflammatory bowel diseases: The next therapeutic frontier. Transl. Res..

[110] Prochnicki T., Latz E. (2017). Inflammasomes on the Crossroads of Innate Immune Recognition and Metabolic Control. Cell Metab..

[111] Zhen Y., Zhang H. (2019). NLRP3 Inflammasome and Inflammatory Bowel Disease. Front. Immunol..

[112] Doedens A.L., Phan A.T., Stradner M.H., Fujimoto J.K., Nguyen J.V., Yang E., Johnson R.S., Goldrath A.W. (2013). Hypoxia-inducible factors enhance the effector responses of CD8(+) T cells to persistent antigen. Nat. Immunol..

[113] Tao J.H., Barbi J., Pan F. (2015). Hypoxia-inducible factors in T lymphocyte differentiation and function. A Review in the Theme: Cellular Responses to Hypoxia. Am. J. Physiol. Cell Physiol..

[114] Palazon A., Tyrakis P.A., Macias D., Velica P., Rundqvist H., Fitzpatrick S., Vojnovic N., Phan A.T., Loman N., Hedenfalk I. (2017). An HIF-1alpha/VEGF-A Axis in Cytotoxic T Cells Regulates Tumor Progression. Cancer Cell.

[115] Mecklenburgh K.I., Walmsley S.R., Cowburn A.S., Wiesener M., Reed B.J., Upton P.D., Deighton J., Greening A.P., Chilvers E.R. (2002). Involvement of a ferroprotein sensor in hypoxia-mediated inhibition of neutrophil apoptosis. Blood.

[116] Kong T., Eltzschig H.K., Karhausen J., Colgan S.P., Shelley C.S. (2004). Leukocyte adhesion during hypoxia is mediated by HIF-1-dependent induction of beta2 integrin gene expression. Proc. Natl. Acad. Sci. USA.

[117] Suzuki M., Inauen W., Kvietys P.R., Grisham M.B., Meininger C., Schelling M.E., Granger H.J., Granger D.N. (1989). Superoxide mediates reperfusion-induced leukocyte-endothelial cell interactions. Am. J. Physiol..

[118] Walmsley S.R., Chilvers E.R., Thompson A.A., Vaughan K., Marriott H.M., Parker L.C., Shaw G., Parmar S., Schneider M., Sabroe I. (2011). Prolyl hydroxylase 3 (PHD3) is essential for hypoxic regulation of neutrophilic inflammation in humans and mice. J. Clin. Investig..

[119] Linfert D., Chowdhry T., Rabb H. (2009). Lymphocytes and ischemia-reperfusion injury. Transplant. Rev..

[120] Zimmerman B.J., Granger D.N. (1990). Reperfusion-induced leukocyte infiltration: Role of elastase. Am. J. Physiol..

[121] Hickey M.J., Kubes P. (2009). Intravascular immunity: The host-pathogen encounter in blood vessels. Nat. Rev. Immunol..

[122] Fluck K., Breves G., Fandrey J., Winning S. (2016). Hypoxia-inducible factor 1 in dendritic cells is crucial for the activation of protective regulatory T cells in murine colitis. Mucosal Immunol..

[123] Kohler T., Reizis B., Johnson R.S., Weighardt H., Forster I. (2012). Influence of hypoxia-inducible factor 1alpha on dendritic cell differentiation and migration. Eur. J. Immunol..

[124] Kuhlicke J., Frick J.S., Morote-Garcia J.C., Rosenberger P., Eltzschig H.K. (2007). Hypoxia inducible factor (HIF)-1 coordinates induction of Toll-like receptors TLR2 and TLR6 during hypoxia. PLoS ONE.

[125] Wobben R., Husecken Y., Lodewick C., Gibbert K., Fandrey J., Winning S. (2013). Role of hypoxia inducible factor-1alpha for interferon synthesis in mouse dendritic cells. Biol. Chem..

[126] Mills E.L., O’Neill L.A. (2016). Reprogramming mitochondrial metabolism in macrophages as an anti-inflammatory signal. Eur. J. Immunol..

[127] Barrero C.A., Datta P.K., Sen S., Deshmane S., Amini S., Khalili K., Merali S. (2013). HIV-1 Vpr modulates macrophage metabolic pathways: A SILAC-based quantitative analysis. PLoS ONE.

[128] Guo X., Zhu Z., Zhang W., Meng X., Zhu Y., Han P., Zhou X., Hu Y., Wang R. (2017). Nuclear translocation of HIF-1alpha induced by influenza A (H1N1) infection is critical to the production of proinflammatory cytokines. Emerg. Microbes Infect..

[129] Staples K.J., Sotoodehnejadnematalahi F., Pearson H., Frankenberger M., Francescut L., Ziegler-Heitbrock L., Burke B. (2011). Monocyte-derived macrophages matured under prolonged hypoxia transcriptionally up-regulate HIF-1alpha mRNA. Immunobiology.

[130] Tannahill G.M., Curtis A.M., Adamik J., Palsson-McDermott E.M., McGettrick A.F., Goel G., Frezza C., Bernard N.J., Kelly B., Foley N.H. (2013). Succinate is an inflammatory signal that induces IL-1beta through HIF-1alpha. Nature.

[131] Filippi M.D. (2019). Neutrophil transendothelial migration: Updates and new perspectives. Blood.

[132] Gayle J., Jones S.L., Argenzio R.A., Blikslager A.T. (2002). Neutrophils increase paracellular permeability of restituted ischemic-injured porcine ileum. Surgery.

[133] Campbell E.L., Bruyninckx W.J., Kelly C.J., Glover L.E., McNamee E.N., Bowers B.E., Bayless A.J., Scully M., Saeedi B.J., Golden-Mason L. (2014). Transmigrating neutrophils shape the mucosal microenvironment through localized oxygen depletion to influence resolution of inflammation. Immunity.

[134] Harrois A., Baudry N., Huet O., Kato H., Lohez M., Ziol M., Duranteau J., Vicaut E. (2013). Synergistic deleterious effect of hypoxemia and hypovolemia on microcirculation in intestinal villi*. Crit. Care Med..

[135] Schwartz B.G., Kloner R.A. (2012). Coronary no reflow. J. Mol. Cell Cardiol..

[136] del Zoppo G.J., Schmid-Schonbein G.W., Mori E., Copeland B.R., Chang C.M. (1991). Polymorphonuclear leukocytes occlude capillaries following middle cerebral artery occlusion and reperfusion in baboons. Stroke.

[137] Hernandez L.A., Grisham M.B., Twohig B., Arfors K.E., Harlan J.M., Granger D.N. (1987). Role of neutrophils in ischemia-reperfusion-induced microvascular injury. Am. J. Physiol..

[138] Biancheri P., Di Sabatino A., Corazza G.R., MacDonald T.T. (2013). Proteases and the gut barrier. Cell Tissue Res..

[139] Maronek M., Marafini I., Gardlik R., Link R., Troncone E., Monteleone G. (2021). Metalloproteinases in Inflammatory Bowel Diseases. J. Inflamm. Res..

[140] Cui N., Hu M., Khalil R.A. (2017). Biochemical and Biological Attributes of Matrix Metalloproteinases. Prog. Mol. Biol. Transl. Sci..

[141] Dejonckheere E., Vandenbroucke R.E., Libert C. (2011). Matrix metalloproteinases as drug targets in ischemia/reperfusion injury. Drug Discov. Today.

[142] Ito K., Kitajima Y., Kai K., Matsufuji S., Yamada K., Egawa N., Kitagawa H., Okuyama K., Tanaka T., Noshiro H. (2021). Matrix metalloproteinase-1 expression is regulated by HIF-1-dependent and epigenetic mechanisms and serves a tumor-suppressive role in gastric cancer progression. Int. J. Oncol..

[143] Zhu S., Zhou Y., Wang L., Zhang J., Wu H., Xiong J., Zhang J., Tian Y., Wang C., Wu H. (2011). Transcriptional upregulation of MT2-MMP in response to hypoxia is promoted by HIF-1alpha in cancer cells. Mol. Carcinog..

[144] Huang C.H., Yang W.H., Chang S.Y., Tai S.K., Tzeng C.H., Kao J.Y., Wu K.J., Yang M.H. (2009). Regulation of membrane-type 4 matrix metalloproteinase by SLUG contributes to hypoxia-mediated metastasis. Neoplasia.

[145] Rajagopalan S., Meng X.P., Ramasamy S., Harrison D.G., Galis Z.S. (1996). Reactive oxygen species produced by macrophage-derived foam cells regulate the activity of vascular matrix metalloproteinases in vitro. Implications for atherosclerotic plaque stability. J. Clin. Investig..

[146] Shah S.V., Baricos W.H., Basci A. (1987). Degradation of human glomerular basement membrane by stimulated neutrophils. Activation of a metalloproteinase(s) by reactive oxygen metabolites. J. Clin. Investig..

[147] Weiss S.J., Peppin G., Ortiz X., Ragsdale C., Test S.T. (1985). Oxidative autoactivation of latent collagenase by human neutrophils. Science.

[148] Haglund U., Bulkley G.B., Granger D.N. (1987). On the pathophysiology of intestinal ischemic injury. Clinical review. Acta Chir. Scand..

[149] Lima A., Bakker J. (2005). Noninvasive monitoring of peripheral perfusion. Intensive Care Med..

[150] Sowa M.G., Kohlenberg E., Payette J.R., Leonardi L., Levasseur M.A., Riley C.B. (2006). Detecting Intestinal Ischemia Using near Infrared Spectroscopy. J. Near Infrared Spectrosc..

[151] Fiddian-Green R.G., Baker S. (1987). Predictive value of the stomach wall pH for complications after cardiac operations: Comparison with other monitoring. Crit. Care Med..

